# Supplementary figures and images for: Towards quantitative viromics for both double-stranded and single-stranded DNA viruses
Source: PeerJ. 2016 Dec 8;4:e2777. doi: 10.7717/peerj.2777 (PMC5168678; doi:10.7717/peerj.2777)

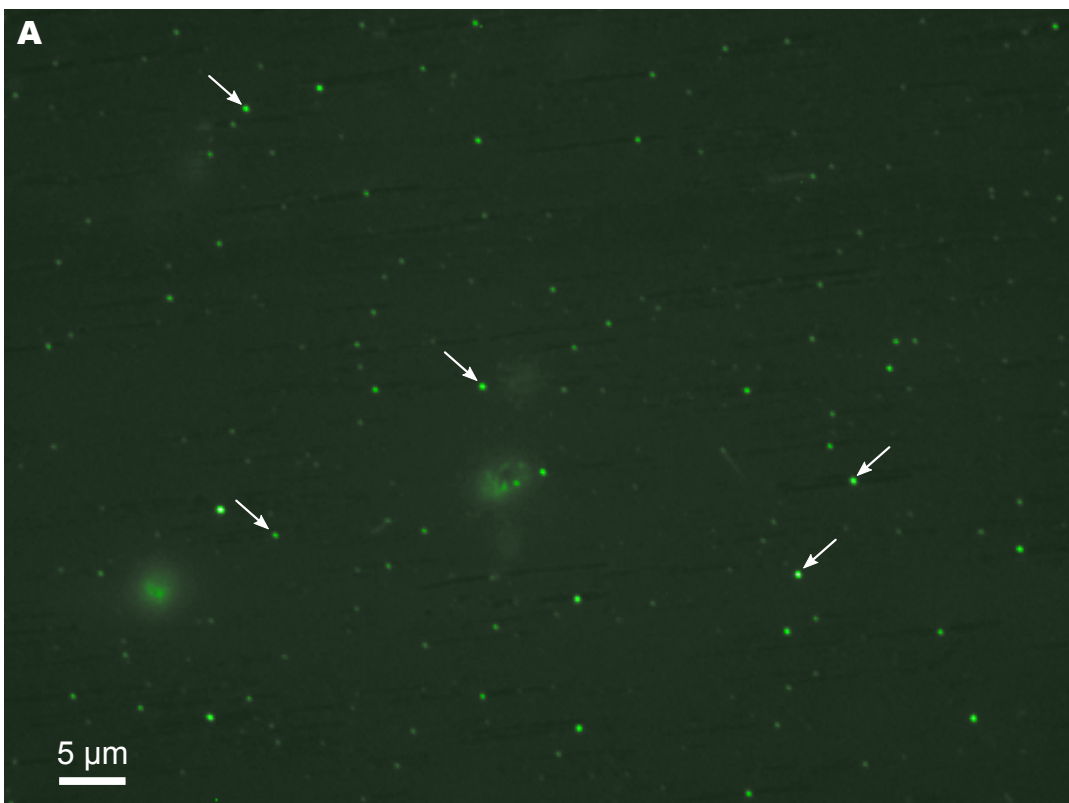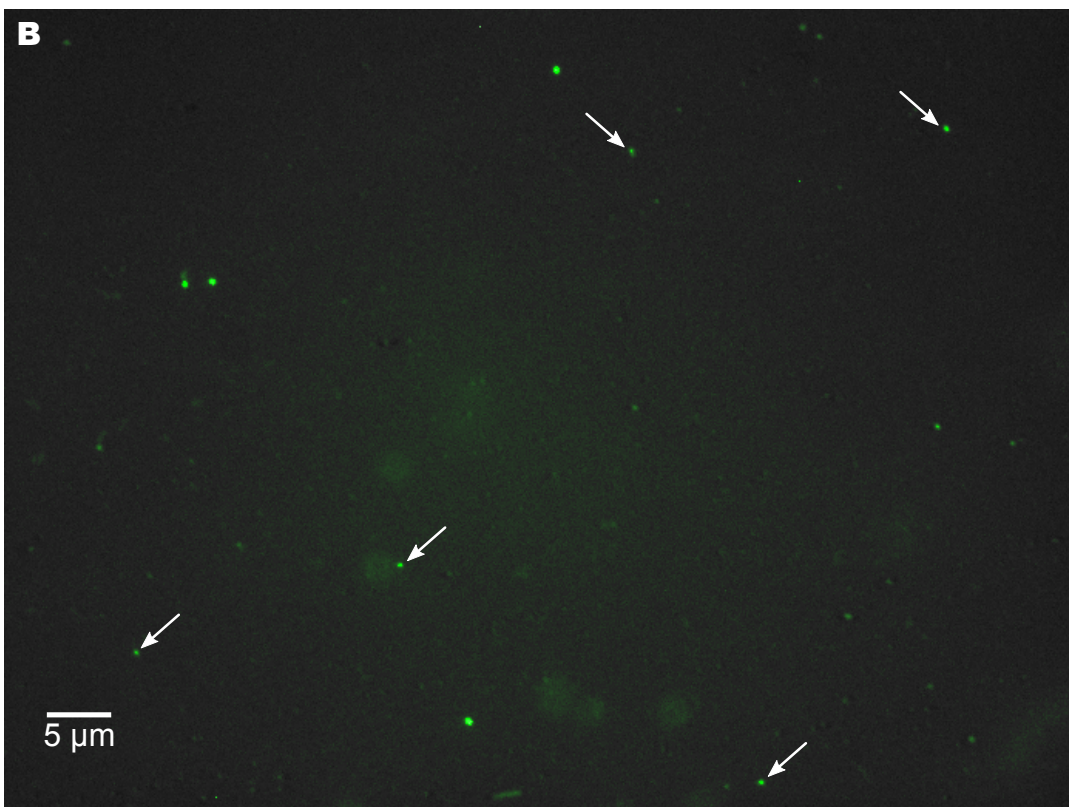

Supplement: Figure S1 — Examples of spots counted as individual viruses are indicated with an arrow (5 for each panel). [file peerj-04-2777-s002.pdf]

Coverage Coefficient of Variation (CV) of dsDNA genomes across viromes

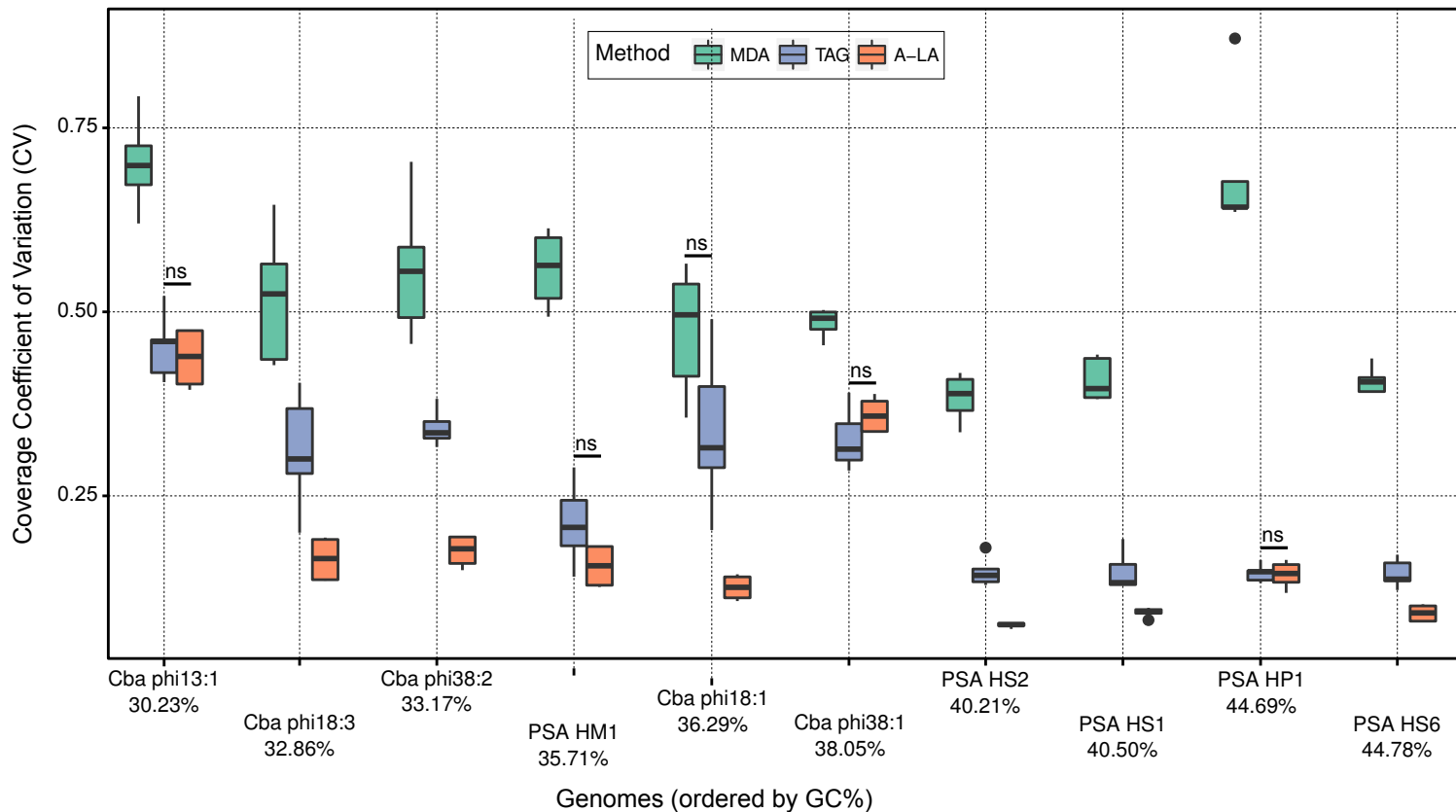

Supplement: Figure S3 — The coverage was estimated for each genome on sliding windows of 1,000bp separated by a 100bp step. The variability of coverage was then estimated for each sample and each genome by calculating the coverage coefficient of variation (standard deviation divided by average). On the x-axis, genomes are ordered according to their overall GC content. All pairs of distributions are significantly different (Wilcoxon two-sided test) except for the ones indicated as “ns”. [file peerj-04-2777-s004.pdf]
