# Supplementary material for: Towards quantitative viromics for both double-stranded and single-stranded DNA viruses
Source: PeerJ. 2016 Dec 8;4:e2777. doi: 10.7717/peerj.2777 (PMC5168678; doi:10.7717/peerj.2777)
Supplement: Figure S2 — The relative abundance of each dsDNA viral genome within the dsDNA viral community (i.e., excluding read mapped to ssDNA viruses) was estimated for each virus in each sample. These relative abundance values are plotted for grouped by sample (left panel), or grouped by genome (right panel). Sample methods (MDA, TAG or A-LA) are indicated via coloring of the boxplot (left panel) or dot (right panel). Genomes with relative abundance significantly different between TAG and A-LA libraries (Wilcoxon two-sided test) are indicated with a star above the boxplot (right panel). [file peerj-04-2777-s003.pdf]

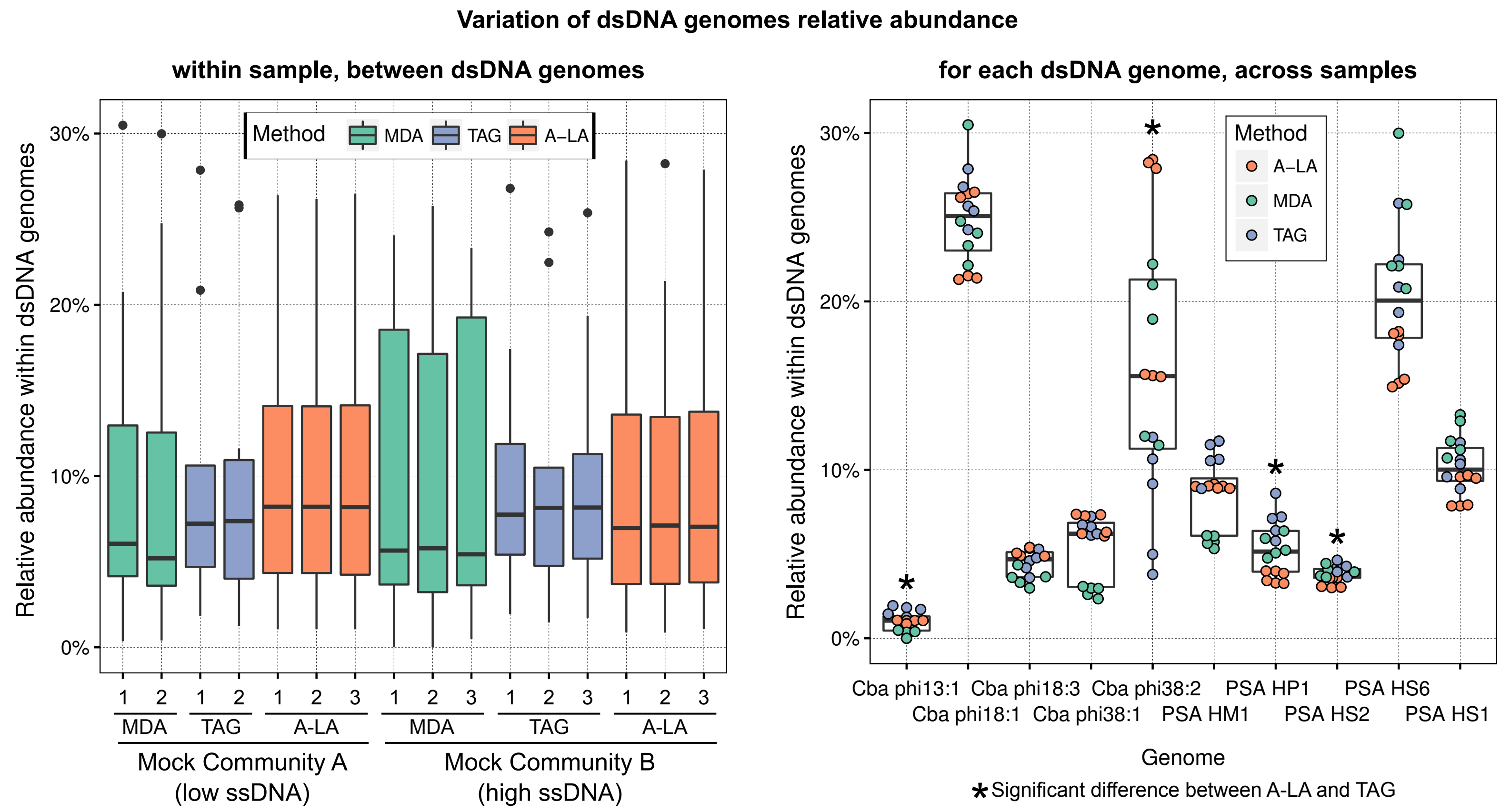
Variation of dsDNA genomes relative abundance
within sample, between dsDNA genomes
for each dsDNA genome, across samples
Method
MDA
TAG
A–LA
Relative abundance within dsDNA genomes
30%
20%
10%
0%
1 2 1 2 1 2 3 1 2 3 1 2 3 1 2 3
MDA TAG A-LA MDA TAG A-LA
Mock Community A
(low ssDNA)
Mock Community B
(high ssDNA)
Cba phi13:1
Cba phi18:1
Cba phi18:3
Cba phi38:1
Cba phi38:2
PSA HM1
PSA HP1
PSA HS2
PSA HS6
PSA HS1
Genome
* Significant difference between A-LA and TAG
